# Study on the Skincare Effects of Red Rice Fermented by *Aspergillus oryzae* In Vitro

**DOI:** 10.3390/molecules29092066

**Published:** 2024-04-30

**Authors:** Mo Chen, Yi Sun, Le Zhu, Lingyu Li, Ya Zhao

**Affiliations:** Shanghai Jahwa United Co., Ltd., Shanghai 200082, China; chenmo@jahwa.com.cn (M.C.); sunyi@jahwa.com.cn (Y.S.); zhule@jahwa.com.cn (L.Z.); lilingyu@jahwa.com.cn (L.L.)

**Keywords:** red ice, *Aspergillus oryzae*, skincare, anti-aging, moisturizing, whitening

## Abstract

Red rice, a variety of pigmented grain, serves dual purposes as both a food and medicinal resource. In recent years, we have witnessed an increasing interest in the dermatological benefits of fermented rice extracts, particularly their whitening and hydrating effects. However, data on the skincare advantages derived from fermenting red rice with *Aspergillus oryzae* remain sparse. This study utilized red rice as a substrate for fermentation by *Aspergillus oryzae*, producing a substance known as red rice *Aspergillus oryzae* fermentation (RRFA). We conducted a preliminary analysis of RRFA’s composition followed by an evaluation of its skincare potential through various in vitro tests. Our objective was to develop a safe and highly effective skincare component for potential cosmetic applications. RRFA’s constituents were assessed using high-performance liquid chromatography (HPLC), Kjeldahl nitrogen determination, the phenol-sulfuric acid method, and enzyme-linked immunosorbent assay (ELISA). We employed human dermal fibroblasts (FB) to assess RRFA’s anti-aging and antioxidative properties, immortalized keratinocytes (HaCaT cells) and 3D epidermal models to examine its moisturizing and reparative capabilities, and human primary melanocytes (MCs) to study its effects on skin lightening. Our findings revealed that RRFA encompasses several bioactive compounds beneficial for skin health. RRFA can significantly promote the proliferation of FB cells. And it markedly enhances the mRNA expression of ECM-related anti-aging genes and reduces reactive oxygen species production. Furthermore, RRFA significantly boosts the expression of Aquaporin 3 (AQP3), Filaggrin (FLG), and Hyaluronan Synthase 1 (HAS1) mRNA, alongside elevating moisture levels in a 3D epidermal model. Increases were also observed in the mRNA expression of Claudin 1 (CLDN1), Involucrin (IVL), and Zonula Occludens-1 (ZO-1) in keratinocytes. Additionally, RRFA demonstrated an inhibitory effect on melanin synthesis. Collectively, RRFA contains diverse ingredients which are beneficial for skin health and showcases multifaceted skincare effects in terms of anti-aging, antioxidant, moisturizing, repairing, and whitening capabilities in vitro, highlighting its potential for future cosmetic applications.

## 1. Introduction

As the economy grows and living standards improve, consumers’ focus on health has heightened, leading to an increased awareness of skincare. Currently, the most sought-after features in skincare products are anti-aging, antioxidant, and whitening properties [1,2]. Correspondingly, there is a growing scrutiny regarding both the effectiveness and safety of skincare ingredients.

Rice, which is known for its beneficial constituents such as phenolic compounds, betaine, and squalene [3], has demonstrated notable anti-aging [4], anti-inflammatory [5], whitening [6], photoprotective [7], and moisturizing benefits, leading to its widespread use in skin and haircare products [8]. Red rice, a vibrant variety of colored rice, has a long history in China as a treasured agricultural and medicinal resource. Its distinctive red hue stems from proanthocyanidins [9]. This nutrient-dense rice is packed with proteins, amino acids, vitamins, trace elements, and functional ingredients [10]. Recent research has underscored the potential of red rice in novel applications, such as functional beer production [11]. Additionally, components like red rice bran and seed coating have shown promise in alleviating conditions like non-alcoholic fatty liver disease by reducing inflammation, managing oxidative stress, and modulating gut flora [12,13]. However, the cosmetic and skincare industry has yet to fully tap into red rice’s potential.

The trend of plant fermentation is gaining traction in the cosmetic industry. Fermented extracts derived from natural botanicals and microbial cultures are proving effective in enhancing the skin surface microecology and boosting skin’s inherent physiological functions [14]. *Aspergillus oryzae*, a common species of filamentous fungi, belongs to the genus *Aspergillus*, but unlike *Aspergillus aflatoxin*, it does not secrete carcinogenic aflatoxins. Metabolic products are widely used in medicines, cosmetics, agriculture, and other fields. Research, like that by Hahm et al., has shown that wheat peptone fermented with *Aspergillus oryzae* can boost human keratinocytes’ proliferation and hydration by activating p44/42 MAPK [15]. Similarly, fermentation of Angelica sinensis with *Aspergillus oryzae* enhances skin’s barrier properties, making it a potential ingredient for managing conditions like atopic dermatitis (AD) [16].

This study utilizes *Aspergillus oryzae* for liquid fermentation of red rice, exploring the skincare benefits of the resultant ferment filtrate through various in vitro tests. Given that fibroblasts play a crucial role in dermal health and their diminishing functionality is a primary aging marker, this research focuses on these cells to assess anti-aging effects [17,18]. Additionally, we investigate key structural proteins in the extracellular matrix (ECM) such as Type I and III collagen and elastin, which are crucial for skin rejuvenation [19,20]. Type I collagen (COL1) is the main component in the dermis. Proteins, arranged on fibers, produce strength and resistance; type III collagen (COL3) usually accompanies COL1 and is closely related to skin regeneration [21]; elastin determines the elasticity and softness of the skin, and is sensitive to physical and chemical reactions such as light. Flow cytometry helps evaluate the antioxidative properties of the red rice ferment filtrate (RRFA) against UVA-induced reactive oxygen species. Furthermore, this study looks into RRFA’s hydrating and reparative properties on keratinocytes and its potential whitening effects on melanocytes via melanin content analysis.

## 2. Results

### 2.1. Composition Analysis of RRFA

The components of RRFA were analyzed, and the results were shown in Table 1. It mainly contains total polyphenols, total proteins, total sugars, and polypeptides. Gamma-aminobutyric acid, VB3 and ceramide were also detected.

### 2.2. Effect of RRFA on Cell Proliferation

**Figure 1:**
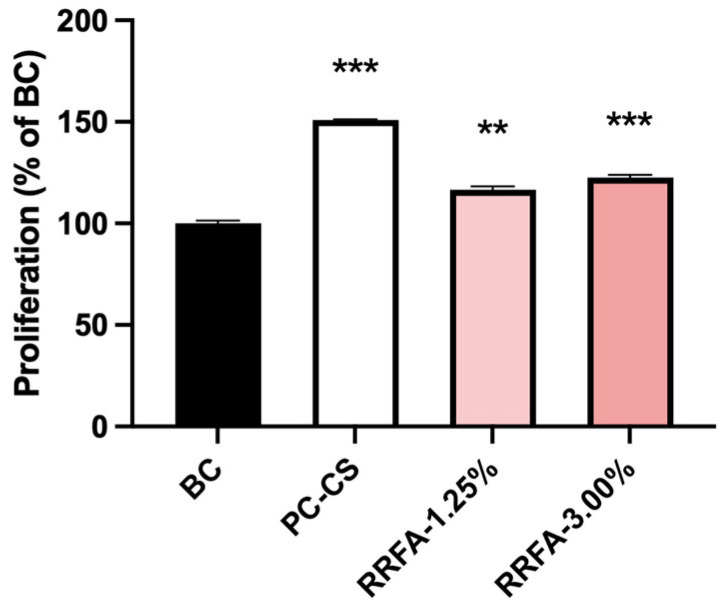
The effect of RRFA on fibroblast proliferation was determined by CCK-8. Data shown are means ± SD (*n* = 6). BC: negative control group treated with DMEM; PC-CS: positive control group treated with 10% CS. Compared with BC, *p* < 0.01 is represented as **, and *p* < 0.001 is represented as ***.

Taking into account future practical applications in formula systems, 3% and 1.25% concentrations were selected as the maximum amounts that could be added. As shown in Figure 1, RRFA enhanced the proliferation potential of FB cells, as evidenced by CCK8. Compared with the BC group, the proliferation rate of the PC group was at 150.87 ± 1.14%. The cell viability significantly increased at 1.25% and 3% concentration (116.63% ± 6.04% and 112.64% ± 4.65%), respectively.

### 2.3. Effect of RRFA on the Expression of Type I Collagen, Type III Collagen, and Elastin mRNA in FB

**Figure 2:**
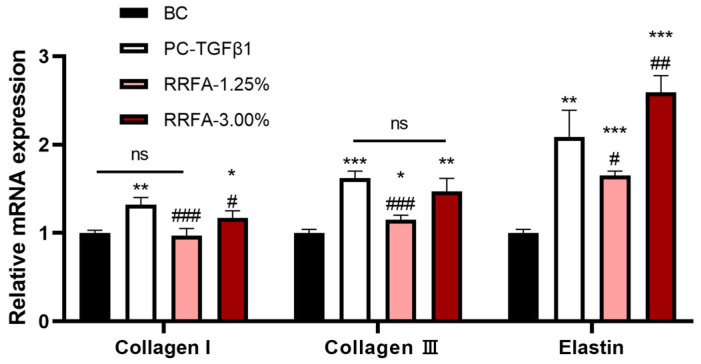
The effect of RRFA on the ECM anti-aging genes of dermal fibroblasts was determined by qRT-PCR. Data shown are mean ± SD (*n* = 3). BC: negative control group treated with DMEM; PC-TGFβ1: positive control group treated with 100 ng/mL TGF-β1. Compared with BC, *p* < 0.05 is represented as *, *p* < 0.01 is represented as **, and *p* < 0.001 is represented as ***. Compared with PC-TGFβ1, *p* < 0.05 is represented as #, *p* < 0.01 is represented as ##, and *p* < 0.001 is represented as ###. And ns represented no difference between the two groups, *p* > 0.05.

Based on the cytotoxicity of fibroblasts and the application of the product, we selected 1.25% and 3% concentrations for testing. As shown in Figure 2, compared with the BC group, RRFA at 3% concentration significantly increased the mRNA levels of COL1, COL3 and elastin genes, with upregulation rates of 17%, 47%, and 159%, respectively; RRFA at 1.25% concentration significantly increased the mRNA levels of COL3 and elastin genes, with upregulation rates of 15% and 65%, respectively. These observations indicate that RRFA increases the gene expression of anti-aging-related proteins in the extracellular matrix of dermal fibroblasts.

### 2.4. Antioxidant Effect of RRFA

**Figure 3:**
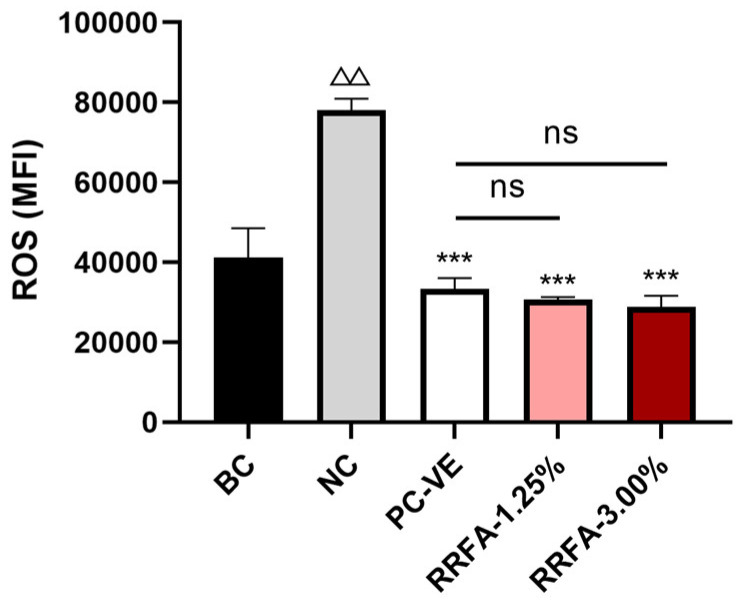
The effect of RRFA on ROS content in UVA-irradiated dermal fibroblasts was determined by flow cytometry. Data shown are mean ± SD (*n* = 3). BC: blank control group treated with DMEM; NC: negative control group treated with 30 J/cm^2^ UVA irradiation and DMEM; PC: positive control group treated with 30 J/cm^2^ UVA irradiation and 0.05% VE. Compared with BC, *p* < 0.01 is represented as △△. Compared with NC, *p* < 0.001 is represented by ***. And ns represented no difference between the two groups, *p* > 0.05.

We studied the antioxidant activity of RRFA by measuring reactive oxygen species (ROS). As shown in Figure 3, ROS detection results showed that after dermal fibroblasts received UVA irradiation of 30 J/cm^2^ (NC group), the ROS content increased significantly compared with that of the BC group (*p* < 0.01). Compared with the NC group, incubation with RRFA at 3% concentration and 1.25% concentration significantly inhibited ROS content, with inhibition rates of 63.04% and 60.82%, respectively (*p* < 0.001). There was no significant difference with the PC group (*p* > 0.05). The above results indicate that RRFA can play an antioxidant role by inhibiting the generation of ROS caused by UVA radiation.

### 2.5. Effect of RRFA on AQP3 and FLG Protein Content

**Figure 4:**
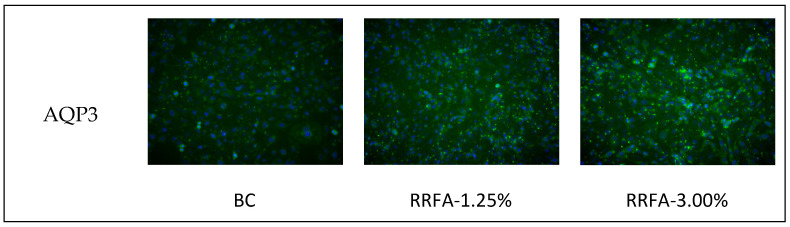

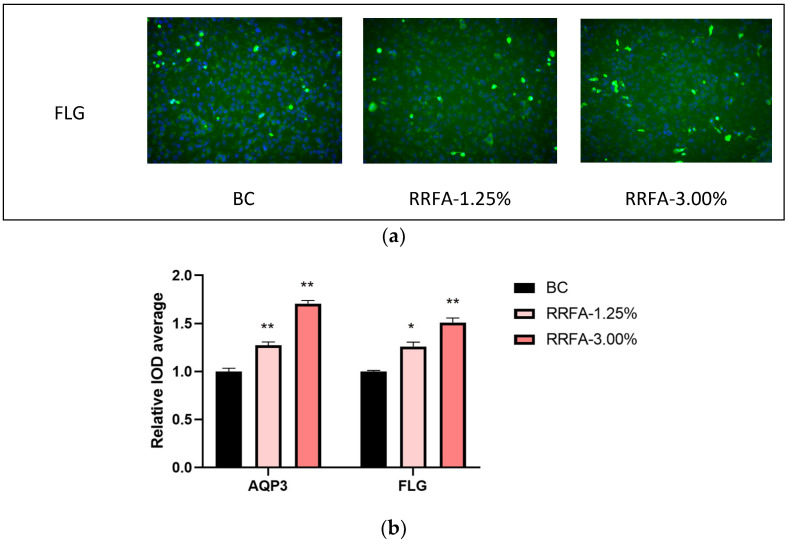
Effect of RRFA on AQP3 and FLG protein expression in keratinocytes. (**a**): Immunofluorescence staining results of AQP3 and FLG in each group; (**b**): relative IOD average of AQP3 and FLG in each group. Data shown are mean ± SD (*n* = 3). BC: blank control group treated with DMEM. Compared with BC, *p* < 0.05 is represented as *, *p* < 0.01 is represented as **.

Aquaporin 3 (AQP3), filaggrin (FLG) plays a key role in various processes involved in keratinocyte function, including hydration, water retention, and barrier repair [22,23]. We investigated the effects of RRFA on expression levels of FLG and AQP3 in human keratinocytes by cellular immunofluorescence. Concentrations of 3% and 1.25% were chosen for the tests. These two concentrations are safe concentrations in HaCat cells. The immunofluorescence staining results of RRFA are shown in Figure 4a (green fluorescence represents protein; blue fluorescence represents cell nucleus), and the relative IOD value is shown in Figure 4b. Compared with the BC group, RRFA significantly increased the AQP3 protein content in keratinocytes at concentrations of 1.25% and 3%, with improvement rates of 28% and 71%, respectively. The effect and relative IOD value of RRFA on FLG protein showed that at concentrations of 1.25% and 3%, the expression of FLG protein was significantly increased, with improvement rates of 26% and 51%, respectively.

### 2.6. Effects of RRFA on the mRNA Expression of HAS1, CLDN1, IVL and ZO-1 in HaCaT Cells

**Figure 5:**
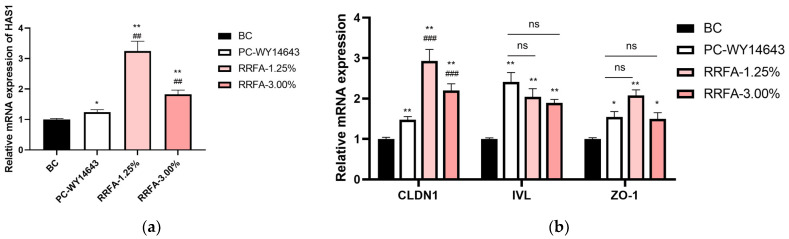
Effect of RRFA on HAS1, CLDN1, IVL and ZO-1 gene expression in HaCaT. Data shown are mean ± SD (*n* = 3). (**a**): effect of RRFA on HAS1. BC: blank control group treated with DMEM. PC-WY4643: positive control treated with 50 μM WY4643. Compared with BC, *p* < 0.05 is represented as *, *p* < 0.01 is represented as **. Compared with PC-WY4643, *p* < 0.01 is represented as ## (**b**): effect of RRFA on CLDN1, IVL and ZO-1. Compared with BC, *p* < 0.05 is represented as *, *p* < 0.01 is represented as **. Compared with PC-WY4643, *p* < 0.001 is represented as ###. And ns represented no difference between the two groups, *p* > 0.05.

Hyaluronic acid is widely present in the extracellular matrix and plays a role in water retention and antioxidant properties [24]. Concentrations of 3% and 1.25% were chosen for the tests. As shown in Figure 5a, compared with the BC group, RRFA at two concentrations could significantly increase the expression of HAS1 mRNA in HaCaT cells, with the upregulation rates being 83.00% and 225.00%, respectively. Tight junctions are an important component of physical barriers and occur mostly in the epidermal layer of the skin, on the apical side of the intercellular space between adjacent cells. Tight junctions are composed of different types of transmembrane proteins and intracellular cytoplasmic proteins, among which CLDN1 and ZO-1 are the key tight junction proteins [25]. Endothelial protein IVL is a marker protein for keratinocyte differentiation and is mainly expressed in the upper layer and granular layer of spinal cells. IVL is located in the outer layer of the keratinocyte envelope, covalently binds to ceramide containing hydroxyl OH, connects the lipid matrix and keratinocytes, and plays a barrier repair role [26].

As shown in Figure 5b, compared with the BC group, RRFA could significantly increase the gene expression of CLDN1 and ZO-1 at concentrations of 3% and 1.25%. Compared with the BC group, RRFA significantly increased IVL gene expression at concentrations of 3% and 1.25%, with the upregulation rates being 89.00%, 104.00%, respectively.

### 2.7. Effects of RRFA on Skin Moisture Content

**Figure 6:**
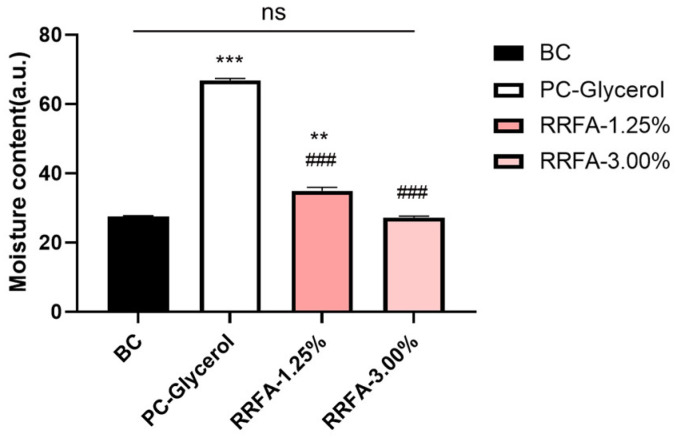
Effect of RRFA on water content of 3D skin models. Data shown are mean ± SD (*n* = 3). BC: blank control group treated with EpiGrowth culture medium; PC-Glycerol: positive control group treated with 20% glycerol. Compared with BC, *p* < 0.01 is represented as **, and *p* < 0.001 is represented as ***. Compared with PC, *p* < 0.001 is represented as ###. And ns represented no difference between the two groups, *p* > 0.05.

Based on the 3D epidermal skin model (EpiKutis^®^, Biocell, Guangzhou, China), the moisturizing effect can be evaluated by directly detecting changes in the skin moisture content of the epidermal skin model after administration. As can be seen from Figure 6, compared with the BC group, the water content of the PC group was significantly increased by 142.96% after incubation with 20% glycerol for 24 h (*p* < 0.001). The water content was significantly increased at 1.25% concentration with an improvement of 26.93% (*p* < 0.01), while there was no significant change in water content at 3% concentration.

### 2.8. The Whitening Effect of RRFA

**Figure 7:**
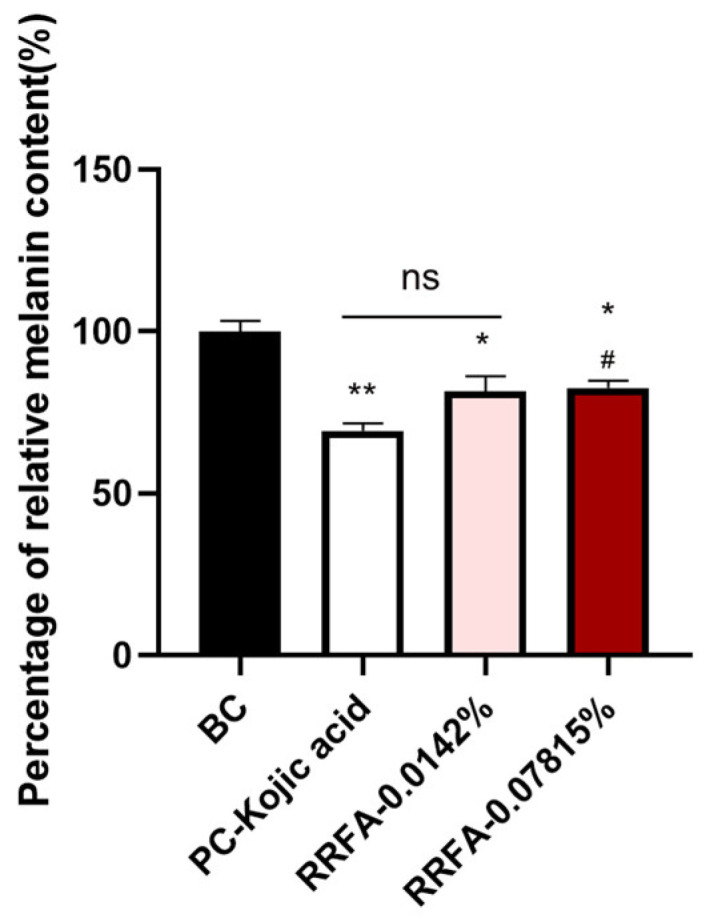
Effect of RRFA on melanin content in human melanocytes. Data shown are mean ± SD (*n* = 3). BC: blank control group treated with DMEM; PC-Kojic acid: positive control group treated with 42.67 μg/mL kojic acid. Compared with BC, *p* < 0.05 is represented as *, *p* < 0.01 is represented as **. Compared with PC, *p* < 0.05 is represented as #. And ns represented no difference between the two groups, *p* > 0.05.

Cytotoxicity is different in different cells. 0.07851% is the maximum non-toxic dose for human melanocytes. A safe concentration for human melanocytes was selected for testing. Also, in combination with the added application of the formulation, a concentration of 0.0142% was selected for testing. As shown in Figure 7, RRFA significantly decreased the melanin content at concentrations of 0.0142% and 0.07815% (*p* < 0.05), and the inhibitory rates were 18.38% and 17.40%, respectively. This suggested that RRFA potentially provides a whitening effect.

## 3. Discussion

After fermenting red rice using *Aspergillus oryzae*, we discovered that red rice aspergillus ferment (RRFA) comprises various compounds which are beneficial for beauty and skincare, including significant amounts of glutamic acid and tyrosine. Studies indicate that polyglutamic acid may moisturize more effectively than hyaluronic acid, while also possessing promising antioxidant properties and skin-whitening capabilities [27]. Peptides, which form over 80% of the total protein in RRFA, have received increasing recognition for their vital skincare benefits in recent years. Additionally, higher polyphenol content in RRFA likely contributes to its superior antioxidant effects. Notably, γ-aminobutyric acid, vitamin B3 (niacin), and ceramides have also been identified within RRFA. These compounds are essential for reinforcing the skin barrier, enhancing skin brightness, decelerating aging, and promoting whitening, making them highly sought-after in skincare products [28,29,30].

This correlation between specific compounds and their skincare efficacies suggests potential strategies for enhancing these ingredients in future formulations. As individuals age, and due to environmental factors such as UV exposure, the activity of skin fibroblasts declines, and the collagen fibers they produce diminish. This reduction leads to decreased skin elasticity, thinning skin, and increased looseness, amplifying wrinkles and accelerating skin aging. RRFA has demonstrated effectiveness in improving the extracellular matrix (ECM), particularly in promoting cell proliferation and genes related to collagen and elastin production. Enhancing its potency through increased concentration and other related techniques might reduce its required dosage in formulations.

Under oxidative stress, the production of reactive oxygen species (ROS) surges, altering membrane lipids, proteins, and nucleic acids. This oxidative harm is intricately linked to aging [31]. RRFA’s ability to inhibit ROS production in dermal fibroblasts further underscores its anti-aging skincare benefits.

RRFA enhances various moisturizing and barrier-related proteins or genes, highlighting its potential for skin hydration and barrier preservation. Nevertheless, our findings indicate that RRFA significantly boosts water content at a 1.25% concentration yet shows no substantial improvement at a higher concentration of 3%. This pattern mirrors changes in the mRNA levels of HAS1, but contrasts with trends in the protein levels of AQP3 and FLG. It is presumed that this could be due to varying evaluation dimensions; moreover, determining whether the upregulation of the HAS1 gene plays a central role in increasing water content necessitates further investigation.

Melanin provides crucial protection against the detrimental effects of UV radiation and other environmental stressors [32]. However, excessive melanin production can lead to skin issues like hyperpigmentation, resulting in dull skin or spots. Melanin is produced by melanocytes in the basal layer of the epidermis and transferred to keratinocytes via dendritic structures. It is then reorganized and distributed as the keratinocytes differentiate, moving upwards and eventually accumulating in the stratum corneum, which contributes to visible pigmentation [33]. Most advanced whitening agents focus on inhibiting tyrosinase activity, promoting tyrosinase protein degradation via the proteasome, or blocking the removal of melanosomes from melanosomes by deactivating protease-activated receptor 2 (PAR2) on the plasma membrane of keratinocytes [34]. At the same time, multiple factors influence melanin synthesis, some of which serve as supplementary metrics for assessing the whitening effectiveness of skincare and beauty products, such as the inhibition of reactive oxygen species, which can directly or indirectly enhance melanocyte activity and melanin production [35].

This study preliminarily confirms RRFA’s impact on melanin levels. Given its inhibitory effect on ROS, it is tentatively suggested that RRFA possesses potential whitening properties. However, whether RRFA can inhibit tyrosinase activity or reduces melanin through the suppression of oxygen free radicals and the downregulation of tyrosinase remains unaddressed and unclear. Additionally, the correlation between its whitening effect and its high content of the whitening agent VB3 requires further validation [36].

Our research also observed a phenomenon in which higher concentrations do not necessarily equate to higher efficacy. For instance, in water content tests, lower concentrations more effectively increased hydration. It is speculated that there may be a dose dependency within a specific concentration range. As RRFA is still an initial extract composed of multiple components, further purification and testing are planned to isolate active ingredients and examine the relationship between concentration and effectiveness. Additionally, integrating product applications, the cosmetic benefits of RRFA should be further validated through human or clinical trials.

## 4. Materials and Methods

### 4.1. Materials

Seventeen kinds of amino acid were purchased from Shanghai Anpel (Shanghai, China). Gallic acid, glucose, gamma-aminobutyric acid, and VB3 were purchased from Shanghai Yuanye Bio-Technology (Shanghai, China). Dulbecco’s modified Eagle’s medium (DMEM), DF-12 medium, fetal bovine serum (FBS), calf serum (CS), phosphate-buffered saline (PBS), and penicillin–streptomycin were purchased from Gibco (Carlsbad, CA, USA). EpiGrowth medium was purchased from Guangdong Biocell (Guangzhou, China). A CCK-8 Test Kit was purchased from Dojindo (Kumamoto, Japan). DMSO, WY14643 and VE were purchased from Sigma (Saint Louis, MO, USA). WY14643 and VE were purchased from Sigma (Saint Louis, MO, USA). RNAiso Plus, a reverse transcription kit, and fluorescent dye were purchased from Accurate Biology Co., Ltd. (Changsha, China). ROS kit and a DCFH-DA probe were purchased from Beyotime (Shanghai, China). TGF-β1 was purchased from Peprotech (Rocky Hill, NJ, USA). Primary antibody and secondary antibody were purchased from Abcam (Cambridge, UK).

### 4.2. The Preparation Procedure of RRFA

After the red rice was crushed, water was added and heated to 50 °C, both amylase and glucoamylase were added for 1 h, and then *Aspergillus oryzae* was added for 36~168 h. After sterilization and centrifuge filtration, an appropriate amount of activated carbon powder was added, then we performed the centrifugal filtration again. Finally, RRFA was obtained through a 0.45 μm microporous filter membrane.

### 4.3. Cell Viability

The experiment set up a negative control group (NC), a positive control group (PC), and a sample group (RRFA). The NC group and PC group were, respectively, combined with 100 μL DMEM and 10% CS and incubated for 24 h. In the RRFA group, two concentration gradients were set (100 μL of 1.25% and 3% RRFA were added, respectively) and 24 h of incubation occurred. After 24 h, the culture medium was discarded, we added 100 μL DMEM (without FBS) containing 10% CCK8 working solution to each well and incubated the resulting mixture at 37 °C for 2 h. A microplate reader was used to measure the absorbance value at 450 nm (BioTek Epoch Microplate Reader, Winooski, VT, USA). The results were expressed as percentages relative to the NC group.

### 4.4. Component Analysis of RRFA

Referring to GB/T 30987-2020 [37], GB 5009.8-2016 [38], QB/T 4587-2013 [39], GB 5009.89-2016 [40], we used high-performance liquid chromatography to detect the contents of free amino acids, glucose, γ-aminobutyric acid, and vitamin B3 in RRFA. The phenol sulfuric acid method was used to detect the total sugar content in RRFA [41]. Referring to GB 5009.5-2016 [42], the protein content in RRFA was determined by the Kjeldahl method. Referring to T/AHFIA 005-2018 [43], the total polyphenol content in RRFA was determined spectrophotometrically. The double-antibody sandwich method was used to determine the ceramide content in RRFA [44].

### 4.5. qRT-PCR Analysis

We seeded cells into a 6-well plate at a seeding density of 2 × 10^5^ cells/well and then incubated them overnight in an incubator (37 °C, 5%CO_2_). According to the test grouping, when the cell plating rate in the 6-well plate reached 40% to 60%, drug administration was carried out in groups. The dosage per well was 2 mL, and each group had 3 duplicate wells. After administration, the 6-well plate was placed in an incubator (37 °C, 5%CO_2_) for 24 h. After 24 h, we discarded the old solution, washed the wells twice with PBS, added 1 mL RNAiso Plus to each well, lysed the cells by pipetting, and collected the samples. RNA was extracted, reverse-transcribed into cDNA, and fluorescent quantitative PCR detection was performed, and the results were calculated using the 2-ΔΔCT method. The primers are shown in Table 2.

### 4.6. Cellular Reactive Oxygen Species (ROS) Detection Assay

Human dermal fibroblasts were seeded into a 6-well plate at a seeding density of 2 × 10^5^ cells/well and incubated overnight in an incubator (37 °C, 5%CO_2_). According to the test grouping, when the cell plating rate in the 6-well plate reached 40% to 60%, drug administration was carried out in groups. The dosage of each well was 2 mL, and each group had 3 duplicate wells in an incubator (37 °C, 5%CO_2_) and was incubated for 24 h. The NC, PC groups, and sample groups were irradiated with UVA at 30 J/cm^2^ and placed in an incubator (37 °C, 5%CO_2_) to continue culturing for 24 h. After irradiation, we directly washed the cells in each well three times with PBS, added 1 mL of 10 μM DCFH-DA probe to each well, and incubated the resulting mixture in an incubator (37 °C, 5%CO_2_) for 30 min. Then, we discarded the DCFH-DA probe. The culture medium of DA was washed three times with PBS. After digesting the cells with trypsin (0.25%), the cells were washed once with PBS. A certain amount of fresh PBS was added, and the mean fluorescence intensity (MFI) of ROS was detected by flow cytometry.

### 4.7. Cell Immunofluorescence Staining

HaCat cells were seeded into a 24-well plate at a density of 1 × 10^5^ cells/well and incubated overnight in an incubator (37 °C, 5%CO_2_). When the cell plating rate in the 24-well plate reached 40% to 60%, we discarded the original cell culture medium and performed drug administration in groups. In the RRFA experimental group, 1.0 mL of working solution containing 0.5%, 1.25%, and 3% RRFA was added to each well. In the BC group, 1.0 mL of cell culture medium was added to each well and 3 duplicate wells were set up. Then, we placed them in an incubator (37 °C, 5%CO_2_) and continued culturing for 24 h. Later we discarded the supernatant, rinsed the cells three times with PBS, and performed routine immunofluorescence staining. The main steps involved fixing, blocking, adding primary antibody, adding secondary antibody, counterstaining with DAPI, and then taking pictures using a fluorescence microscope. The results of the fluorescence intensity of AQP3 and FLG were quantitatively analyzed using Image Pro Plus software (6.0).

### 4.8. Three-Dimensional Epidermal Model Moisture Content Test

Based on the 3D epidermal skin model (EpiKutis^®^, Biocell, Guangzhou, China), the BC group did not undergo any treatment. We added 20% glycerol to the PC group, and the sample group evenly distributed samples of the corresponding concentrations on the surface of the model and placed them in an incubator (37 °C, 5%CO_2_) for 24 h. After 24 h, we cleaned the surface of the model, cut off the model ring, and measured the water content using a Corneometer^®^ CM 825 skin moisture tester (CK, Köln, Germany). Each model was measured three times and the average value was taken.

### 4.9. Melanin Content Test Based on Human Melanocytes

Inoculated human melanocytes were placed in a 6-well plate at a seeding density of 1.5 × 10^5^ cells/well and incubated overnight in an incubator (37 °C, 5%CO_2_). When the cell plating rate in the 6-well plate reached 50% to 60%, drug administration was carried out in groups. The dosage per well was 2 mL. Each group had 3 duplicate wells and was incubated in an incubator (37 °C, 5%CO_2_) for 24 h. The melanocytes were digested with 0.25% trypsin, collected in a 1.5 mL centrifuge tube, and centrifuged at 10,000 r/min for 10 min, and the supernatant was discarded. We added 1 mL of 1 mol/L NaOH aqueous solution containing 10% DMSO to the centrifuge tube and heated it in an 80 °C water bath for 40 min. After thermal incubation, we pipetted 200 μL of supernatant into a 96-well plate and read the OD value at 405 nm (BioTek Epoch Microplate Reader, Winooski, VT, USA).

## 5. Statistical Analysis

GraphPad Prism (San Diego, CA, USA) was used for graphing, and the results were expressed as the mean ± SD. Comparisons between groups were analyzed using *t*-test statistics. The statistical analyses were all two-tailed. *p* < 0.05 was considered a significant difference, and *p* < 0.01 was considered a highly significant difference.

## 6. Conclusions

This study showed that RRFA obtained from red rice via *Aspergillus oryzae* fermentation had many skincare effects such as anti-aging, antioxidation, moisturizing, repairing, and whitening in vitro. These results suggest its potential application value in beauty and skincare. Currently, RRFA has been preliminarily used in anti-aging repair cream.

## Figures and Tables

**Table 1:** Components of RRFA.

Ingredient	Content
Total protein	0.27 wt%
Total polyphenols	0.01 wt%
Total sugar	9.9 wt%
Peptide	0.22 wt%
Gamma-aminobutyric acid	13.21 mg/L
VB3	17.61 mg/L
Ceramide	330.66 pg/mL

**Table 2:** List of primers for qRT-PCR.

Name	Forward Primer (5′-3′) Reverse Primer (5′-3′)
Collagen I	GTGGCAGTGATGGAAGTGTG AGGACCAGCGTTACCAACAG
Collagen III	ACCAGGAGCTAACGGTCTCA TCTGATCCAGGGTTTCCATC
Elastin	ACCCCTGACTCACGACCTCA CGCTCCCCTCTTGTTTCCTT
HAS1	ACTCGGACACAAGGTTGGAC TTAGGAAGCTGACCCAGGAG
CLDN1	GCATGAAGTGTATGAAGTGCTTGGA CCATACCATGCTGTGGCAACTAA
IVL	CCACTTATTTCGGGTCCGCT CTGAGGTTGGGATTGGGGTC
ZO-1	CTCAGCCTGTGAGGCGTAGT GCTGTGCTCTTACTGTGGCA

## Data Availability

Human melanocytes (lot number: MC210817), keratinocytes (lot number: Ep230129), fibroblasts (lot number: Fb19052002, Fb20081902) were all purchased from Guangdong Biocell (Guangzhou, China).

## References

[1] Ferreira M.S., Magalhães M.C., Oliveira R., Sousa-Lobo J.M., Almeida I.F. (2021). Trends in the Use of Botanicals in Anti-Aging Cosmetics. Molecules.

[2] Ribeiro A.S., Estanqueiro M., Oliveira M.B., Sousa Lobo J.M. (2015). Main benefits and applicability of plant extracts in skin care products. Cosmetics.

[3] Zamil D.H., Khan R.M., Braun T.L., Nawas Z.Y. (2022). Dermatological uses of rice products: Trend or true?. J. Cosmet. Dermatol..

[4] Kanlayavattanakul M., Lourith N., Chaikul P. (2016). Jasmine rice panicle: A safe and efficient natural ingredient for skin aging treatments. J. Ethnopharmacol..

[5] Palungwachira P., Tancharoen S., Phruksaniyom C., Klungsaeng S., Srichan R., Kikuchi K., Nararatwanchai T. (2019). Antioxidant and Anti-Inflammatory Properties of Anthocyanins Extracted from *Oryza sativa*, L. in Primary Dermal Fibroblasts. Oxidative Med. Cell. Longev..

[6] Chung S.Y., Seo Y.K., Park J.M., Seo M.J., Park J.K., Kim J.W., Park C.S. (2009). Fermented rice bran downregulates MITF expression and leads to inhibition of alpha-MSH-induced melanogenesis in B16F1 melanoma. Biosci. Biotechnol. Biochem..

[7] Seo Y.-K., Jung S.-H., Song K.-Y., Park J.-K. (2010). Anti-photoaging effect of fermented rice bran extract on UV-induced normal skin fibroblasts. Eur. Food Res. Technol..

[8] Kovach M.J., Sweeney M.T., McCouch S.R. (2007). New insights into the history of rice domestication. Trends Genet..

[9] Piazzon A., Forte M., Nardini M. (2010). Characterization of phenolics content and antioxidant activity of different beer types. J. Agric. Food Chem..

[10] Li W., Fan Z., Fawen W., Yan L., Haifeng Q., Hui Z., Xiguang Q. (2019). The healthy benefits and applications of red rice. Food Mach..

[11] Mehra R., Kumar H., Kumar N., Kaushik R. (2020). Red rice conjugated with barley and rhododendron extracts for new variant of beer. J. Food Sci. Technol..

[12] Munkong N., Somnuk S., Jantarach N., Ruxsanawet K., Nuntaboon P., Kanjoo V., Yoysungnoen B. (2023). Red Rice Bran Extract Alleviates Hih-Fat Diet-Induced Non-Alcoholic Fatty Liver Disease and Dyslipidemia in Mice. Nutrients.

[13] Chen Y., Zhao Z., Guo S., Li Y., Yin H., Tian L., Cheng G., Li Y. (2023). Red Rice Seed Coat Targeting SPHK2 Ameliorated Alcoholic Liver Disease via Restored Intestinal Barrier and Improved Gut Microbiota in Mice. Nutrients.

[14] Dou J., Feng N., Guo F., Chen Z., Liang J., Wang T., Guo X., Xu Z. (2023). Applications of Probiotic Constituents in Cosmetics. Molecules.

[15] Hahm K.M., Park S.H., Oh S.W., Kim J.H., Yeom H.S., Lee H.J., Yang S., Cho J.Y., Park J.O., Lee J. (2021). *Aspergillus oryzae*-Fermented Wheat Peptone Enhances the Potential of Proliferation and Hydration of Human Keratinocytes through Activation of p44/42 MAPK. Molecules.

[16] Ha C.W., Sohn E.H., Kim S.H., Jang S., Park M.R., Kim Y.K., Bae I.Y. (2022). Fermented Angelicae tenussimae with *Aspergillus oryzae* Improves Skin Barrier Properties, Moisturizing, and Anti-Inflammatory Responses. Int. J. Mol. Sci..

[17] Zhang M. (2022). Research Progress on the Mechanism of Fibroblasts in Skin Aging. Adv. Clin. Med..

[18] Xu Y. (2023). The relationship between the molecular mechanism of fibroblast aging and skin aging and research progress. Chin. Aesthetic Med..

[19] Binic I., Lazarevic V., Ljubenovic M., Mojsa J., Sokolovic D. (2013). Skin ageing: Natural weapons and strategies. Evid.-Based Complement. Altern. Med..

[20] Xiong Z.M., O’Donovan M., Sun L., Choi J.Y., Ren M., Cao K. (2017). Anti-Aging Potentials of Methylene Blue for Human Skin Longevity. Sci. Rep..

[21] Harris A.K., Stopak D., Wild P. (1981). Fibroblast traction as a mechanism for collagen morphogenesis. Nature.

[22] Bollag W.B., Aitkens L., White J., Hyndman K.A. (2020). Aquaporin-3 in the epidermis: More than skin deep. Am. J. Physiol. Cell Physiol..

[23] Kezic S., O’Regan G.M., Yau N., Sandilands A., Chen H., Campbell L.E., Kroboth K., Watson R., Rowland M., McLean W.H. (2011). Levels of filaggrin degradation products are influenced by both filaggrin genotype and atopic dermatitis severity. Allergy.

[24] Chen H., Hossain M.A., Kim J.H., Cho J.Y. (2021). Kahweol Exerts Skin Moisturizing Activities by Upregulating STAT1 Activity. Int. J. Mol. Sci..

[25] Kuo I.H., Carpenter-Mendini A., Yoshida T., McGirt L.Y., Ivanov A.I., Barnes K.C., Gallo R.L., Borkowski A.W., Yamasaki K., Leung D.Y. (2013). Activation of epidermal toll-like receptor 2 enhances tight junction function: Implications for atopic dermatitis and skin barrier repair. J. Investig. Dermatol..

[26] Kanwal S., Singh S.K., Soman S.P., Choudhury S., Kumari P., Ram P.K., Garg S.K. (2021). Expression of barrier proteins in the skin lesions and inflammatory cytokines in peripheral blood mononuclear cells of atopic dogs. Sci. Rep..

[27] Wang L., Chen S., Yu B. (2022). Poly-γ-glutamic acid: Recent achievements, diverse applications and future perspectives. Trends Food Sci. Technol..

[28] Uehara E., Hokazono H., Hida M., Sasaki T., Yoshioka H., Matsuo N. (2017). GABA promotes elastin synthesis and elastin fiber formation in normal human dermal fibroblasts (HDFs). Biosci. Biotechnol. Biochem..

[29] Chen A.C., Damian D.L. (2014). Nicotinamide and the skin. Australas. J. Dermatol..

[30] Uchida Y., Park K. (2021). Ceramides in Skin Health and Disease: An Update. Am. J. Clin. Dermatol..

[31] Jadoon S., Karim S., Bin Asad M.H., Akram M.R., Khan A.K., Malik A., Chen C., Murtaza G. (2015). Anti-Aging Potential of Phytoextract Loaded-Pharmaceutical Creams for Human Skin Cell Longetivity. Oxid. Med. Cell Longev..

[32] Slominski R.M., Sarna T., Płonka P.M., Raman C., Brożyna A.A., Slominski A.T. (2022). Melanoma, Melanin, and Melanogenesis: The Yin and Yang Relationship. Front. Oncol..

[33] Ye X., Zhu P. (2016). Research progress on melanin synthesis and whitening products. J. East China Norm. Univ. (Nat. Sci. Ed.).

[34] Ren Q., Wu H., Jin J. (2021). Cosmetic plant raw materials (IV)—Research and development of plant whitening raw materials that inhibit the melanin synthesis signaling pathway. Dly. Chem. Ind..

[35] Cheng Q., Gao L., Deng F., Zhong Y. (2019). Application progress of antioxidants in the cosmetics industry. Dly. Chem. Sci..

[36] Hakozaki T., Minwalla L., Zhuang J., Chhoa M., Matsubara A., Miyamoto K., Greatens A., Hillebrand G.G., Bissett D.L., Boissy R.E. (2002). The effect of niacinamide on reducing cutaneous pigmentation and suppression of melanosome transfer. Br. J. Dermatol..

[37] (2020). Determination of Free Amino Acids in Plant.

[38] (2016). Determination of Fructose, Glucose, Sucrose, Maltose and Lactose in Foods.

[39] (2013). Gamma. Aminobutyric Acid.

[40] (2016). Determination of Niacin and Nicotinamide in Foods.

[41] (2009). Determination of Total Saccharide in Edible Mushroom.

[42] (2016). National Food Safety Standard Determination of Protein in Foods.

[43] (2018). Determination of Total Polyphenol Content in Plant Extracts and their Products Spectrophotometry Method.

[44] Xie J.P., Zhang M.L., Liu Z. (2002). Research progress on ceramides and their analysis and separation techniques. Fine Chem..

